# Nanostructured Materials and Advanced Processes for Application in Water Purification

**DOI:** 10.3390/nano13040654

**Published:** 2023-02-07

**Authors:** Christos A. Aggelopoulos

**Affiliations:** Laboratory of Cold Plasma and Advanced Techniques for Improving Environmental Systems, Institute of Chemical Engineering Sciences, Foundation for Research and Technology Hellas (FORTH/ICE-HT), 26 504 Patras, Greece; caggelop@iceht.forth.gr

Water pollution is a major environmental problem that has a significant impact on human and animal health and the ecosystem. Most pollution is caused by human activities, and pollutants can be categorized into inorganic, organic, biological and radioactive [1]. Thus, the critical issue of improving water quality has several social and economic benefits.

During the last decade, the field of nanotechnology has extensively developed, contributing to a significant impact on water purification. The use of nanomaterials has led to impressive findings in the field of water remediation, with a high efficiency for the removal of various pollutants, cost effectiveness and reusability. Thus, nanostructured materials, due to their unique physicochemical characteristics, such as catalytic activity; high physical, chemical and thermal stability; large specific surface area; high chemical reactivity; strong electron ability, etc., have gained the attention of many researchers. Different classes of nanomaterials can be used in water purification either as adsorbents, photocatalysts and/or antibacterial agents [2,3,4,5]. More specific, nanostructured materials used as adsorbents (nanosorbents), nanoparticles (e.g., Pd, Au, Cu, Fe_3_O_4_, TiO_2_, ZnO, Ag, etc.) and nanocatalytic membrane systems are more efficient and less time-consuming than other treatment methods, environmentally friendly and consume a low amount of energy [4].

This Special Issue focuses on nanostructured materials and their applications in advanced water purification processes. This Special Issue contains fifteen articles and one communication that address the synthesis, modification and regeneration of novel nanomaterials for their applications to remediate water contaminated by various pollutants.

The first paper published in this Special Issue by Xiang et al. [6] illustrates the preparation of Fe_3_O_4_@C nanoparticles for the decolorization of high concentrations of methylene blue. According to the obtained results, an easy method for producing Fe_3_O_4_@C nanoparticles with excellent catalytic reactivity is presented, representing a promising approach for the industrial production of Fe_3_O_4_@C nanoparticles used to treat high concentrations of dyes in wastewater. The second paper by Zia et al. [7] presents the synthesis of silver/iron oxide nanocomposites (Ag/Fe_3_O_4_) for the efficient and specific removal of iodine anions from contaminated water. The findings presented in this study offer a novel method for desalinating radioiodine in various aqueous media. The next paper by Narath et al. [8] proposes a facile green synthetic method for the synthesis of zinc oxide nanoparticles (ZnO NPs) using the bio-template *Cinnamomum tamala* (*C. tamala*) leaves extract. According to the obtained results, the synthesized ZnO NPs exhibit excellent photocatalytic activity against dye molecules, through a green protocol. The fourth paper by Shrestha [9] illustrates the usage of wood dust of *Dalbergia sisoo* (Sisau) derived from activated carbon (AC) as an adsorbent material for the removal of rhodamine B dye from an aqueous solution. According to the author, this study successfully addressed a local problem of wastewater pollution from garment and textile industrial effluents using the locally available agro-waste of *Dalbergia sisoo*. Khan et al. [10] investigated the removal of nano-CuO from pure water and montmorillonite clay (MC) suspensions, using poly aluminum ferric chloride (PAFC) and cationic polyacrylamide (PAM) via the coagulation–flocculation–sedimentation (C/F/S) process. Moreover, PAFC and PAFC/PAM flocculation performance was investigated through a parametric analysis. The findings presented in this paper provide insights into enhanced flocculation and the coagulation of CuO in drinking water containing clay particles. Balarak et al. [11] found that activated porous carbon prepared from Azolla filiculoides (AF) (AFAC) could be a potential low-cost adsorbent of azithromycin (AZM) from aqueous solutions. In the next paper by Liou, Chen and Yang [12], amino-functionalized nanoporous Santa Barbara amorphous-15 (SBA-15) was fabricated by employing sodium silicate as a precursor and successfully applied as a high-efficiency adsorbent for the removal of tannic acid from aqueous media. Shalabayev et al. [13] reported the successful synthesis of CdS nanoparticles using cadmium acetate and sodium sulfide as Cd and S precursors, respectively. Moreover, the effect of using sodium thiosulfate as an additional sulfur precursor was also investigated (combined milling) as well as the antibacterial potential of mechanochemically prepared CdS nanocrystals on reference strains of *E. coli* and *S. aureus*. The results presented, demonstrated that solvent-free and sustainable mechanochemical synthesis can easily produce semiconductor nanocrystals in a multidisciplinary application. In the paper by Peñaranda et al. [14], three different magnetic microreactors based on torus geometries (i.e., one-loop, two-horizontal-loop, and two-vertical-loop microreactors) were designed, manufactured, and tested to increase the enzyme-based transformation of dyes by laccase bio-nanocomposites, improve particle suspension, and promote the interaction of reagents. Taken together, these results indicate that the novel microreactors introduced in this study have the potential to efficiently process wastewaters contaminated with dyes in a continuous mode, with potential for large-scale operations. Primo et al. [15] present two green synthesis routes that were used for the synthesis of Ag/ZnO nanoparticles, using cassava starch as a simple and low-cost effective fuel and aloe vera as a reducing and stabilizing agent. The results indicate that Ag/ZnO nanoparticles synthesized via green chemistry are a promising candidate for the treatment of wastewaters contaminated by bacteria (*E. coli*), due to their easy preparation, low-cost synthesis, and disinfection efficiency. Magro et al. [16] successfully developed sensors based on nanostructured thin films deposited on ceramic substrates with gold-interdigitated electrodes to detect azithromycin, clarithromycin, and erythromycin in concentrations ranging between 10^−15^ M and 10^−5^ M in mineral and river water matrices. Ramírez-Rodríguez et al. [17] proposed a novel method to fabricate a hybrid adsorbent membrane of whey protein isolate (WPI) and polycaprolactone (PCL) by electrospinning, finding promising results for the removal heavy metal ions (e.g., chromium ions) from water. Khan et al. [18] focus on the synthesis of a nanocomposite material (ZnO–CuO/g–C_3_N_4_) using a solution method for the successful extraction of arsenic (III), pointing out that a ZnO–CuO/g–C_3_N_4_ nanocomposite can be a potential candidate for the enhanced removal of arsenic from water reservoirs. Zhang et al. [19], prepared a new catalyst, copper oxide/graphene oxide–diatomaceous earth (CuO/GO-DE) using the ultrasonic impregnation method, which had excellent catalytic activity towards ciprofloxacin degradation. Tóth et al. [20] presented the effect of different phosphate sources on the synthesis and photocatalytic activity of Ag_3_PO_4_ for pollutants degradation. These authors concluded that Ag_3_PO_4_–based materials could be reliably used for the degradation of methyl orange (MO) as they mostly retain their photoactivity during the second recycling test. In the final paper by Giannoulia et al. [21], halloysite nanoclay (HNC) was examined as an adsorbent for the individual and simultaneous removal of antibiotic enrofloxacin (ENRO) and methylene blue (MB) from aqueous solutions, alongside its successful regeneration via cold atmospheric plasma (CAP) bubbling. In addition, CAP bubbling induced chemical modifications on the HNC surface, increasing its adsorption capacity when applied to new adsorption cycles.

## Data Availability

The data presented in this study are available on request from the corresponding author.

## References

[1] Schweitzer L., Noblet J. (2018). Water contamination and pollution. Green Chemistry.

[2] Santhosh C., Velmurugan V., Jacob G., Jeong S.K., Grace A.N., Bhatnagar A. (2016). Role of nanomaterials in water treatment applications: A review. Chem. Eng. J..

[3] Saleem H., Zaidi S.J. (2020). Developments in the application of nanomaterials for water treatment and their impact on the environment. Nanomaterials.

[4] Nasrollahzadeh M., Sajjadi M., Iravani S., Varma R.S. (2021). Green-synthesized nanocatalysts and nanomaterials for water treatment: Current challenges and future perspectives. J. Hazard. Mater..

[5] Xu C., Nasrollahzadeh M., Sajjadi M., Maham M., Luque R., Puente-Santiago A.R. (2019). Benign-by-design nature-inspired nanosystems in biofuels production and catalytic applications. Renew. Sustain. Energy Rev..

[6] Xiang H., Ren G., Zhong Y., Xu D., Zhang Z., Wang X., Yang X. (2021). Fe_3_O_4_@C Nanoparticles Synthesized by In Situ Solid-Phase Method for Removal of Methylene Blue. Nanomaterials.

[7] Zia M.R., Raza M.A., Park S.H., Irfan N., Ahmed R., Park J.E., Mushtaq S. (2021). Removal of Radioactive Iodine Using Silver/Iron Oxide Composite Nanoadsorbents. Nanomaterials.

[8] Narath S., Koroth S.K., Shankar S.S., George B., Mutta V., Wacławek S., Varma R.S. (2021). Cinnamomum tamala leaf extract stabilized zinc oxide nanoparticles: A promising photocatalyst for methylene blue degradation. Nanomaterials.

[9] Shrestha D. (2021). Efficiency of wood-dust of Dalbergia sisoo as low-cost adsorbent for rhodamine-B dye removal. Nanomaterials.

[10] Khan R., Inam M.A., Lee K.H., Channa A.S., Mallah M.A., Wie Y.M., Abbasi M.N. (2021). Synergetic Effect of Organic Flocculant and Montmorillonite Clay on the Removal of Nano-CuO by Coagulation-Flocculation-Sedimentation Process. Nanomaterials.

[11] Balarak D., Mahvi A.H., Shahbaksh S., Wahab M.A., Abdala A. (2021). Adsorptive removal of azithromycin antibiotic from aqueous solution by azolla filiculoides-based activated porous carbon. Nanomaterials.

[12] Liou T.H., Chen G.W., Yang S. (2022). Preparation of Amino-Functionalized Mesoporous SBA-15 Nanoparticles and the Improved Adsorption of Tannic Acid in Wastewater. Nanomaterials.

[13] Shalabayev Z., Baláž M., Khan N., Nurlan Y., Augustyniak A., Daneu N., Burkitbayev M. (2022). Sustainable Synthesis of Cadmium Sulfide, with Applicability in Photocatalysis, Hydrogen Production, and as an Antibacterial Agent, Using Two Mechanochemical Protocols. Nanomaterials.

[14] Peñaranda P.A., Noguera M.J., Florez S.L., Husserl J., Ornelas-Soto N., Cruz J.C., Osma J.F. (2022). Treatment of Wastewater, Phenols and Dyes Using Novel Magnetic Torus Microreactors and Laccase Immobilized on Magnetite Nanoparticles. Nanomaterials.

[15] Primo J.D.O., Horsth D.F., Correa J.D.S., Das A., Bittencourt C., Umek P., Anaissi F.J. (2022). Synthesis and Characterization of Ag/ZnO Nanoparticles for Bacteria Disinfection in Water. Nanomaterials.

[16] Magro C., Moura T., Dionísio J., Ribeiro P.A., Raposo M., Sério S. (2022). Nanostructured Metal Oxide Sensors for Antibiotic Monitoring in Mineral and River Water. Nanomaterials.

[17] Ramírez-Rodríguez L.C., Quintanilla-Carvajal M.X., Mendoza-Castillo D.I., Bonilla-Petriciolet A., Jiménez-Junca C. (2022). Preparation and Characterization of an Electrospun Whey Protein/Polycaprolactone Nanofiber Membrane for Chromium Removal from Water. Nanomaterials.

[18] Khan Q.U., Begum N., Rehman Z.U., Khan A.U., Tahir K., Tag El Din E.S.M., Javed M.S. (2022). Development of Efficient and Recyclable ZnO–CuO/g–C_3_N_4_ Nanocomposite for Enhanced Adsorption of Arsenic from Wastewater. Nanomaterials.

[19] Zhang T., Zhang J., Yu Y., Li J., Zhou Z., Li C. (2022). Synthesis of CuO/GO-DE Catalyst and Its Catalytic Properties and Mechanism on Ciprofloxacin Degradation. Nanomaterials.

[20] Tóth Z.R., Debreczeni D., Gyulavári T., Székely I., Todea M., Kovács G., Hernadi K. (2023). Rapid Synthesis Method of Ag_3_PO_4_ as Reusable Photocatalytically Active Semiconductor. Nanomaterials.

[21] Giannoulia S., Triantaphyllidou I.E., Tekerlekopoulou A.G., Aggelopoulos C.A. (2023). Mechanisms of Individual and Simultaneous Adsorption of Antibiotics and Dyes onto Halloysite Nanoclay and Regeneration of Saturated Adsorbent via Cold Plasma Bubbling. Nanomaterials.

